# Nonparametric Causal Structure Learning in High Dimensions

**DOI:** 10.3390/e24030351

**Published:** 2022-02-28

**Authors:** Shubhadeep Chakraborty, Ali Shojaie

**Affiliations:** Department of Biostatistics, University of Washington, Seattle, WA 98195, USA; deep20@uw.edu

**Keywords:** causal structure learning, consistency, FCI algorithm, high dimensionality, nonparametric testing, PC algorithm

## Abstract

The PC and FCI algorithms are popular constraint-based methods for learning the structure of directed acyclic graphs (DAGs) in the absence and presence of latent and selection variables, respectively. These algorithms (and their order-independent variants, PC-stable and FCI-stable) have been shown to be consistent for learning sparse high-dimensional DAGs based on partial correlations. However, inferring conditional independences from partial correlations is valid if the data are jointly Gaussian or generated from a linear structural equation model—an assumption that may be violated in many applications. To broaden the scope of high-dimensional causal structure learning, we propose nonparametric variants of the PC-stable and FCI-stable algorithms that employ the conditional distance covariance (CdCov) to test for conditional independence relationships. As the key theoretical contribution, we prove that the high-dimensional consistency of the PC-stable and FCI-stable algorithms carry over to general distributions over DAGs when we implement CdCov-based nonparametric tests for conditional independence. Numerical studies demonstrate that our proposed algorithms perform nearly as good as the PC-stable and FCI-stable for Gaussian distributions, and offer advantages in non-Gaussian graphical models.

## 1. Introduction

Directed acyclic graphs (DAGs) are commonly used to represent causal relationships among random variables [1,2,3]. The PC algorithm [3] is the most popular constraint-based method for learning DAGs from observational data under the assumption of causal sufficiency, i.e., when there are no unmeasured common causes and no selection variables. It first estimates the skeleton of a DAG by recursively performing a sequence of conditional independence tests, and then uses the information from the conditional independence relations to partially orient the edges, resulting in a completed partially directed acyclic graph (CPDAG). In Section 2, we provide a review of these and other notions commonly used in the graphical modeling literature that are relevant to our work. In addition, we refer to estimating the CPDAG as structure learning of the underlying DAG throughout the rest of the paper.

Observational studies often involve latent and selection variables, which complicate the causal structure learning problem. Ignoring such unmeasured variables can make the causal inference based on the PC algorithm erroneous; see, e.g., Section 1.2 in [4] for some illustrations. The Fast Causal Inference (FCI) algorithm and its variants [3,4,5,6] utilize similar strategies as the PC algorithm to learn the DAG structure in the presence of latent and selection variables.

Both PC and FCI algorithms adopt a hierarchical search strategy—they recursively perform conditional independence tests given subsets of increasingly larger cardinalities in some appropriate search pool. The PC algorithm is usually order-dependent, in the sense that its output depends on the order in which pairs of adjacent vertices and subsets of their adjacency sets are considered. The FCI algorithm suffers from a similar limitation. To overcome this limitation, Ref. [7] proposed two variants of the PC and FCI algorithms, namely the PC-stable and FCI-stable algorithms that resolve the order dependence at different stages of the algorithms.

In general, testing for conditional independence is a problem of central importance in the causal structure learning. The literature on the PC and FCI algorithms predominantly uses partial correlations to infer conditional independence relations. It is well-known that the characterization of conditional independence by partial correlations, or, in other words, equivalence between conditional independence and zero partial correlations only holds for multivariate normal random variables. Therefore, the high-dimensional consistency results for the PC and FCI algorithms [4,8] are limited to Gaussian graphical models, where the nodes correspond to random variables with a joint Gaussian distribution. Although the Gaussian graphical model is the standard parametric model for continuous data, it may not hold in many real data applications. Although this limitation can be somewhat relaxed by considering linear structural equation models (SEMs) with general noise distributions [9], linear SEMs and joint Gaussianity are essentially equivalent [10]. Moreover, neither approach is appropriate when the observations are categorical, discrete, or are supported on a subset of the real line. In Section 4.3, for example, we present a real application where all the observed variables are categorical, and therefore far from being Gaussian. As an improvement, ref. [11] used rank-based partial correlations to test for conditional independence relations, showing that the high-dimensional consistency of the PC algorithm holds for a broader class of Gaussian copula models. Some nonparametric versions of the PC algorithm have been also proposed in the literature via kernel-based tests for conditional independence [12,13]; however, they lack theoretical justifications of the correctness of the algorithms, and are not studied in high dimensions.

This work aims to broaden the applicability of the PC-stable and FCI-stable algorithms to general distributions by employing a nonparametric test for conditional independence relationships. To this end, we utilize recent developments on dependence metrics that quantify nonlinear and non-monotone dependence between multivariate random variables. More specifically, our work builds on the idea of distance covariance (dCov) proposed by [14] and its extension to conditional distance covariance (CdCov) by [15] as a nonparametric measure of nonlinear and non-monotone conditional independence between two random vectors of arbitrary dimensions given a third. Utilizing this flexibility, we use the conditional distance covariance (CdCov) to test for conditional independence relationships in the sample versions of the PC-stable and FCI-stable algorithms. The resulting algorithms—which, for distinction, are termed *nonPC* and *nonFCI*—facilitate causal structure learning from general distributions over DAGs and are shown to be consistent in sparse high-dimensional settings. We establish the consistency of the proposed algorithms using some moment and tail conditions on the variables, without requiring strict distributional assumptions. To our knowledge, the proposed generalizations of PC/PC-stable or the FCI/FCI-stable algorithms provide the first general nonparametric framework for causal structure learning with theoretical guarantees in high dimensions.

The rest of the paper is organized as follows: In Section 2, we review the relevant background, including preliminaries on graphical modeling (Section 2.1), an outline of the PC-stable and FCI-stable algorithms (Section 2.2) and a brief overview of dCov and CdCov (Section 2.3). The nonparametric version of the PC-stable algorithm is presented in Section 3.1. As a key contribution of the paper, we establish that the algorithm consistently estimates the skeleton and the equivalence class of the underlying sparse high-dimensional DAG in a general nonparametric framework. We then present the nonparametric version of the FCI-stable algorithm in Section 3.2 and establish its consistency in sparse high-dimensional settings. As the FCI involves the adjacency search of the PC algorithm, any improvement on the PC/PC-stable directly carries over to the FCI/FCI-stable as well. In Section 4, we compare the performances of our algorithms with the PC-stable and FCI-stable using both simulated datasets (involving both Gaussian and non-Gaussian examples), as well as a real dataset. These numerical studies clearly demonstrate that nonPC and nonFCI algorithms are comparable with PC-stable and FCI-stable for Gaussian data and offer improvements for non-Gaussian data.

## 2. Background

### 2.1. Preliminaries on Graphical Modeling

We start with introducing some necessary terminologies and background information. Our notations and terminologies follow standard conventions in graphical modeling (see, e.g., [3]). A graph G=(V,E) consists of a vertex set V={1,⋯,p} and an edge set E⊆V×V. In a graphical model, the vertices or nodes are associated with random variables $X_{a}$ for 1≤a≤p. Throughout, we index the nodes by the corresponding random variables. We also allow the edge set *E* of the graph G to contain (a subset of) the following six types of edges: → (*directed*), ↔ (*bidirected*), − (*undirected*), ∘−∘ (*nondirected*), ∘− (*partially undirected*) and ∘→ (*partially directed*). The endpoints of an edge are called marks, which can be tails, arrowheads or circles. A “∘” at the end of an edge indicates it is not known whether an arrowhead should occur at that place. We use the symbol ‘★’ to denote an arbitrary edge mark; for example, the symbol ★→ represents an edge of the type →, ↔ or ∘→ in the graph. A *mixed graph* is a graph containing directed, bidirected and undirected edges. A graph containing only directed edges (→) is called a *directed graph*, one containing only undirected edges (−) is called an *undirected graph*, and one containing directed and undirected edges is called a *partially directed graph*.

The *adjacency set* of a vertex $X_{a}$ in the graph G=(V,E), denoted $\mathrm{adj}(G,X_{a})$, is the set of all vertices in *V* that are adjacent to $X_{a}$, or, in other words, are connected to $X_{a}$ by an edge. The *degree* of a vertex $X_{a}$, $\left|\mathrm{adj}(G,X_{a})\right|$, is defined as the number of vertices adjacent to it. A graph is *complete* if all pairs of vertices in the graph are adjacent. A vertex $X_{b}\in \mathrm{adj}\left(G,X_{a}\right)$ is called a *parent* of $X_{a}$ if $X_{b}\to X_{a}$, a *child* of $X_{a}$ if $X_{a}\to X_{b}$  and a *neighbor* of $X_{a}$ if $X_{a}-X_{b}$. The *skeleton* of the graph G is the undirected graph obtained by replacing all the edges of G by undirected edges, in other words, ignoring all the edge orientations. Three vertices $\langle X_{a},X_{b},X_{c}\rangle$ are called an *unshielded triple* if $X_{a}$ and $X_{b}$ are adjacent, $X_{b}$ and $X_{c}$ are adjacent, but $X_{a}$ and $X_{c}$ are not adjacent. A *path* is a sequence of distinct adjacent vertices. A node $X_{a}$ is an *ancestor* of its *descendent*$X_{b}$, if G contains a directed path $X_{a}\to \cdots \to X_{b}$. A non-endpoint vertex $X_{a}$ on a path is called a collider on the path if both the edges preceding and succeeding it have an arrowhead at $X_{a}$, or, in other words, the path contains $★\;\to X_{a}\leftarrow \;★$. An unshielded triple $\langle X_{a},X_{b},X_{c}\rangle$ is called a *v-structure* if $X_{b}$ is a collider on the path $\langle X_{a},X_{b},X_{c}\rangle$.

A *cycle* occurs in a graph when there is a path from $X_{a}$ to $X_{b}$, and $X_{a}$ and $X_{b}$ are adjacent. A directed path from $X_{a}$ to $X_{b}$ forms a *directed cycle* together with the edge $X_{b}\to X_{a}$, and it forms an *almost directed cycle* together with the edge $X_{b}↔X_{a}$. Three vertices that form a cycle are called a *triangle*. A *directed acyclic graph* (DAG) is a directed graph that does not contain any cycle. A DAG entails conditional independence relationships via a graphical criterion called *d-separation* (Section 1.2.3 in [16]). Two vertices $X_{a}$ and $X_{b}$ that are not adjacent in a DAG G are d-separated in G by a subset $X_{S}\subseteq V\setminus \left\{X_{a},X_{b}\right\}$. A probability distribution *P* on $R^{p}$ is said to be *faithful* with respect to the DAG G if the conditional independence relationships in *P* can be inferred from G using d-separation and vice versa; in other words, $X_{a}⫫X_{b}|X_{S}$ if and only if $X_{a}$ and $X_{b}$ are d-separated in G by $X_{S}$.

A graph that is both (partially) directed and acyclic is called a *partially directed acyclic graph (PDAG)*. DAGs that encode the same set of conditional independence relations form a Markov equivalence class [17]. Two DAGs belong to the same Markov equivalence class if and only if they have the same skeleton and the same v-structures. A Markov equivalence class of DAGs can be uniquely represented by a *completed partially directed acyclic graph (CPDAG)*, which is a PDAG that satisfies the following: (i) $X_{a}\to X_{b}$ in the CPDAG if $X_{a}\to X_{b}$ in every DAG in the Markov equivalence class, and (ii) $X_{a}-X_{b}$ in the CPDAG if the Markov equivalence class contains a DAG in which $X_{a}\to X_{b}$ as well as a DAG in which $X_{a}\leftarrow X_{b}$.

### 2.2. The PC-Stable and FCI-Stable Algorithms

In this section, we provide an outline of the PC/PC-stable and FCI/FCI-stable algorithms. Estimation of the CPDAG by the PC algorithm involves two steps: (1) estimation of the skeleton and separating sets (also called the adjacency search step); and (2) partial orientation of edges; see Algorithms 1 and 2 in [8] for details.

Intuitively, the PC algorithm works as follows. In the first step (the adjacency search step), the algorithm starts with a complete undirected graph Then, for conditioning sets of increasing cardinality, k=0,1,…, the algorithm removed an edge $X_{a}-X_{b}$ if $X_{a}$ and $X_{b}$ are conditionally independent given a subset *S* of size *k* chosen among the current neighbors of nodes *a* and *b*. This process continues up to the order q−1, where *q* is the maximum degree of the underlying DAG. By searching over the neighboring nodes, the algorithm is adaptive and can efficiently infer sparse high-dimensional DAGs, where the sparsity is characterized by the maximum node degree, *q*.

In the presence of latent and selection variables, one needs a generalization of an DAG, called a *maximal ancestral graph* (MAG). A mixed graph is called an *ancestral graph* if it contains no directed or almost directed cycles and no subgraph of the type $X_{a}-X_{b}\leftarrow \;★\;X_{c}$. DAGs form a subset of ancestral graphs. A MAG is an ancestral graph in which every missing edge corresponds to a conditional independence relationship via the m-separation criterion [18], a generalization of the notion of d-separation. Multiple MAGs may represent the same set of conditional independence relations. Such MAGs form a Markov equivalence class which can be represented by a *partial ancestral graph* (PAG) [19]; see [18] for additional details.

Under the faithfulness assumption, the Markov equivalence class of a DAG with latent and selection variables can be learned using the FCI algorithm (e.g., Algorithm 3.1 in [4]), which is a modification of the PC algorithm. The FCI algorithm first employs the adjacency search of the PC algorithm, and then performs additional conditional independence queries because of the presence of latent variables followed by partial orientation of the edges, resulting in an estimated PAG. The FCI algorithm adopts the same hierarchical search strategy as the PC algorithm: It starts with a complete undirected graph and recursively removes edges via conditional independence queries given subsets of increasingly larger cardinalities in some appropriate search pool.

The PC algorithm is usually order-dependent, in the sense that its output depends on the order in which pairs of adjacent vertices and subsets of their adjacency sets are considered. The FCI algorithm suffers from a similar limitation, as it shares the adjacency search step of the PC algorithm as its first step. To overcome this limitation, ref. [7] proposed variants of the PC and FCI algorithms, namely the PC-stable and FCI-stable algorithms that resolve the order dependence at different stages of the algorithms. The basic difference between the PC algorithm and the PC-stable algorithm is that, in the adjacency search step, the latter computes and stores the adjacency sets of all the variables after each new cardinality, k=0,1,…, of the conditioning sets. These stored adjacency sets are then used to search for conditioning sets of this given size *k*. As a consequence, the removal of an edge no longer affects which conditional independence relations need to be checked for other pairs of variables at this given size of the conditioning sets.

We would refer the reader to Appendix A, where we provide in full detail the pseudocodes of the *oracle* versions of the PC-stable and FCI-stable algorithms. In the *oracle* versions of the algorithms, it is assumed that perfect knowledge is available about all the necessary conditional independence relations. As such, conditional independence relations are not estimated from data. Of course, this perfect knowledge is not available in practice. *Sample* versions of the PC-stable and FCI-stable algorithms can be obtained by replacing the conditional independence queries by a suitable test for conditional independence at some pre-specified level. For example, if the variables are jointly Gaussian, one can test for zero partial correlations (see, e.g., [8]). The next subsection is devoted to discussions on nonparametric tests for independence and conditional independence.

### 2.3. Distance Covariance and Conditional Distance Covariance

We start by describing the notation used throughout the paper. We denote by ${∥\cdot ∥}_{p}$ the Euclidean norm of $R^{p}$ and use ∥·∥ when the dimension is clear from the context. We use X⫫Y to denote the independence of *X* and *Y* and use $E_{U}$ to denote expectation with respect to the probability distribution of the random variable *U*. For any set *S*, we denote its cardinality by |S|.

We use the usual asymptotic notation, ‘*O*’ and ‘*o*’, as well as their probabilistic counterparts, $O_{p}$ and $o_{p}$, which denote stochastic boundedness and convergence in probability, respectively. For two sequences of real numbers ${\left\{a_{n}\right\}}_{n=1}^{\infty }$ and ${\left\{b_{n}\right\}}_{n=1}^{\infty }$, $a_{n}≍b_{n}$ if and only if $a_{n}/b_{n}=O\left(1\right)$ and $b_{n}/a_{n}=O\left(1\right)$ as n→∞. We use the symbol “a≲b” to indicate that a≤Cb  for some constant C>0. For a matrix $A={\left(a_{kl}\right)}_{k,l=1}^{n}\in R^{n\times n}$, we denote its determinant by |A| and define its  U-centered version $\tilde{A}={\left({\tilde{a}}_{kl}\right)}_{k,l=1}^{n}$ as

(1) $$\begin{array}{c} {\tilde{a}}_{kl}=\left\{\begin{array}{cc} a_{kl}\;-\;\frac{1}{n-2}\sum_{j=1}^{n}a_{kj}\;-\;\frac{1}{n-2}\sum_{i=1}^{n}a_{il}\;+\;\frac{1}{\left(n-1)(n-2\right)}\sum_{i,j=1}^{n}a_{ij},\; & k\neq l,\\ 0, & k=l, \end{array}\right. \end{array}$$

for k,l=1,⋯,n. We denote the indicator function of any set *A* by 1(A). Finally, we denote the integer part of a∈R by ⌊a⌋.

Ref. [14], in their seminal paper, introduced the notion of distance covariance (dCov, henceforth) to quantify nonlinear and non-monotone dependence between two random vectors of arbitrary dimensions. Consider two random vectors $X\in R^{p}$ and $Y\in R^{q}$ with ${E∥X∥}_{p}<\infty$ and ${E∥Y∥}_{q}<\infty$. The distance covariance between *X* and *Y* is defined as the positive square root of

$$\begin{array}{c} {\mathrm{dCov}}^{2}\left(X,Y\right)=\frac{1}{c_{p}c_{q}}\int_{R^{p+q}}\frac{|f_{X,Y}\left(t,s\right)-f_{X}\left(t\right)f_{Y}{\left(s\right)|}^{2}}{{∥t∥}_{p}^{1+p}\;{∥s∥}_{q}^{1+q}}dtds\; \end{array}$$

where $f_{X}$, $f_{Y}$ and $f_{X,Y}$ are the individual and joint characteristic functions of *X* and *Y*, respectively, and $c_{p}=\pi^{(1+p)/2}/\;\Gamma \left(\left(1+p\right)/2\right)$ is a constant with Γ(·) being the complete gamma function.

The key feature of dCov is that it completely characterizes the independence between two random vectors, or in other words dCov(X,Y)=0 if and only if X⫫Y. According to Remark 3 in [14], dCov can be equivalently expressed as

$$\begin{array}{cc} {\mathrm{dCov}}^{2}\left(X,Y\right)\;=\; & E\;∥X-X^{\prime }{∥}_{p}∥Y-Y^{\prime }{∥}_{q}\;+\;E\;∥X-X^{\prime }{∥}_{p}\;E\;{∥Y-Y^{\prime }∥}_{q}\\ & \;-\;2\;E\;∥X-X^{\prime }{∥}_{p}{∥Y-Y^{\prime \prime }∥}_{q}\;. \end{array}$$

This alternate expression comes handy in constructing V or U-statistic type estimators for the quantity. For an observed random sample ${\left(X_{i},Y_{i}\right)}_{i=1}^{n}$ from the joint distribution of *X* and *Y*, define the distance matrices $d^{X}={\left(d_{ij}^{X}\right)}_{i,j=1}^{n}$ and $d^{Y}={\left(d_{ij}^{Y}\right)}_{i,j=1}^{n}\in R^{n\times n}$, where $d_{ij}^{X}:={∥X_{i}-X_{j}∥}_{p}$ and $d_{ij}^{Y}:={∥Y_{i}-Y_{j}∥}_{q}$. Following the U-centering idea in [20], an unbiased U-statistic type estimator of ${\mathrm{dCov}}^{2}\left(X,Y\right)$ can be expressed as

(2) $$\begin{array}{cc} {\mathrm{dCov}}_{n}^{2}\left(X,Y\right)\; & :=\;\left({\tilde{d}}^{\;X}\cdot \;{\tilde{d}}^{\;Y}\right)\;:=\;\frac{1}{n(n-3)}\sum_{i\neq j}{\tilde{d}}_{ij}^{\;X}{\tilde{d}}_{ij}^{\;Y}\;, \end{array}$$

where ${\tilde{d}}^{\;X}={\left({\tilde{d}}_{ij}^{\;X}\right)}_{i,j=1}^{n}$  and   ${\tilde{d}}^{\;Y}={\left({\tilde{d}}_{ij}^{\;Y}\right)}_{i,j=1}^{n}$ are the  U-centered versions of the matrices $d^{\;X}$ and $d^{\;Y}$, respectively, as defined in (1).

Ref. [15] generalized the notion of dCov and introduced the conditional distance covariance (CdCov, henceforth) as a measure of conditional dependence between two random vectors of arbitrary dimensions given a third. CdCov essentially replaces the characteristic functions used in the definition of dCov by conditional characteristic functions. Consider a third random vector $Z\in R^{r}$ with $E(∥X{∥}_{p}+∥Y{∥}_{q}|Z)<\infty$. Denote by $f_{X,Y|Z}$ the conditional joint characteristic function of *X* and *Y* given *Z*, and by $f_{X|Z}$ and $f_{Y|Z}$ the conditional marginal characteristic functions of *X* and *Y* given *Z*, respectively. Then, CdCov between *X* and *Y* given *Z* is defined as the positive square root of

$$\begin{array}{c} {\mathrm{CdCov}}^{2}\left(X,Y|Z\right)=\frac{1}{c_{p}c_{q}}\int_{R^{p+q}}\frac{|f_{X,Y|Z}\left(t,s\right)-f_{X|Z}\left(t\right)f_{Y|Z}{\left(s\right)|}^{2}}{{∥t∥}_{p}^{1+p}\;{∥s∥}_{q}^{1+q}}dtds. \end{array}$$

The key feature of CdCov is that CdCov(X,Y|Z)=0 almost surely if and only if X⫫Y|Z, which is quite straightforward to see from the definition.

Similar to dCov, an equivalent alternative expression can be established for CdCov that avoids complicated integrations involving conditional characteristic functions. Let ${\left\{W_{i}=\left(X_{i},Y_{i},Z_{i}\right)\right\}}_{i=1}^{n}$ be an i.i.d. sample from the joint distribution of W:=(X,Y,Z). Define $d_{ijkl}:=\left(d_{ij}^{X}+d_{kl}^{X}-d_{ik}^{X}-d_{jl}^{X}\right)\;\left(d_{ij}^{Y}+d_{kl}^{Y}-d_{ik}^{Y}-d_{jl}^{Y}\right)$, which is not symmetric with respect to {i,j,k,l}, and therefore necessitates defining the following symmetric form: $d_{ijkl}^{S}:=d_{ijkl}\;+\;d_{ijlk}\;+\;d_{ilkj}$. Lemma 1 in [15] establishes an equivalent representation of ${\mathrm{CdCov}}^{2}\left(X,Y|Z=z\right)$ as

(3) $$\begin{array}{c} {\mathrm{CdCov}}^{2}\left(X,Y|Z=z\right)\;=\;\frac{1}{12}\;E\;\left[d_{1234}^{S}\;|\;Z_{1}=z,Z_{2}=z,Z_{3}=z,Z_{4}=z\right]\;. \end{array}$$

**Remark** **1.** *In a recent work, [21] explore the connection between conditional independence measures induced by distances on a metric space and reproducing kernels associated with a reproducing kernel Hilbert space (RKHS). They generalize CdCov to arbitrary metric spaces of negative type—termed generalized CdCov (gCdCov)—and develop a kernel-based measure of conditional independence, namely the Hilbert–Schmidt conditional independence criterion (HSCIC). Theorem 1 in their paper establishes an equivalence between gCdCov and HSCIC, or, in other words, between distance and kernel-based measures of conditional independence.*

For $w\in R^{r}$, let $K_{H}\left(w\right):={\left|H\right|}^{-1}\;K\left(H^{-1}w\right)$ be a kernel function, where *H* is the diagonal matrix diag(h,⋯,h) determined by a bandwidth parameter *h*. $K_{H}$ is typically considered to be the Gaussian kernel $K_{H}\left(w\right)={\left(2\pi \right)}^{-\frac{r}{2}}\;{\left|H\right|}^{-1}\;\exp\left(-\frac{1}{2}w^{T}H^{-2}w\right)$, where $w\in R^{r}$.

Let $K_{iu}:=K_{H}\left(Z_{i}-Z_{u}\right)={\left|H\right|}^{-1}\;K\left(H^{-1}\left(Z_{i}-Z_{u}\right)\right)$ and $K_{i}\left(Z\right):=K_{H}\left(Z-Z_{i}\right)$ for 1≤i,u≤n. Then, by virtue of the equivalent representation of CdCov in (3), a V-statistic type estimator of ${\mathrm{CdCov}}^{2}\left(X,Y|Z\right)$ can be constructed as

(4) $$\begin{array}{c} {\mathrm{CdCov}}_{n}^{2}\left(X,Y|Z\right)\;:=\;\sum_{i,j,k,l}\frac{K_{i}\left(Z\right)\;K_{j}\left(Z\right)\;K_{k}\left(Z\right)\;K_{l}\left(Z\right)}{12\;{\left(\sum_{i=1}^{n}K_{i}\left(Z\right)\right)}^{4}}\;d_{ijkl}^{S}\;. \end{array}$$

Under certain regularity conditions, Theorem 4 in [15] shows that, conditioned on *Z*, ${\mathrm{CdCov}}_{n}^{2}\left(X,Y|Z\right)\overset{P}{⟶}{\mathrm{CdCov}}^{2}\left(X,Y|Z\right)$ as n→∞.

## 3. Methodology and Theory

### 3.1. The Nonparametric PC Algorithm in High Dimensions

To obtain a measure of conditional independence between *X* and *Y* given *Z* that is free of *Z*, we define

(5) $$\begin{array}{c} \rho_{0}^{*}\;\left(X,Y|Z\right)\;:=\;E\;\left[{\mathrm{CdCov}}_{n}^{2}\left(X,Y|Z\right)\right]\;. \end{array}$$

Clearly, $\rho_{0}^{*}\;\left(X,Y|Z\right)=0$ if and only if X⫫Y|Z. Consider a plug-in estimate of $\rho_{0}^{*}\;\left(X,Y|Z\right)$ as

(6) $$\begin{array}{c} \begin{array}{cc} \; & {\hat{\rho }}^{\;*}\left(X,Y|Z\right)\;:=\;\frac{1}{n}\sum_{u=1}^{n}{\mathrm{CdCov}}_{n}^{2}\left(X,Y|Z_{u}\right)\;\;=\;\frac{1}{n}\sum_{u=1}^{n}\Delta_{i,j,k,l;u}\;\\ \mathrm{where}\; & \Delta_{i,j,k,l;u}\;:=\;\sum_{i,j,k,l}\frac{K_{iu}\;K_{ju}\;K_{ku}\;K_{lu}}{12\;{\left(\sum_{i=1}^{n}K_{iu}\right)}^{4}}\;d_{ijkl}^{S}\;. \end{array} \end{array}$$

We reject $H_{0}:X⫫Y|Z$  vs  HA:X⫫Y|Z at level α∈(0,1) if  ${\hat{\rho }}^{\;*}\left(X,Y|Z\right)>\xi_{\alpha }$, for a suitably chosen threshold $\xi_{\alpha }$. In Appendix A, we present a local bootstrap procedure for choosing $\xi_{\alpha }$ in practice, which is also used in our numerical studies. Henceforth, we will often denote $\rho_{0}^{*}\;\left(X,Y|Z\right)$ and ${\hat{\rho }}^{\;*}\left(X,Y|Z\right)$ simply by $\rho_{0}^{*}$ and ${\hat{\rho }}^{\;*}$ respectively for notational simplicity, whenever there is no confusion.

In view of the complete characterization of conditional independence by $\rho_{0}^{*}$, we propose testing for conditional independence relations nonparametrically in the sample version of the PC-stable algorithm based on $\rho_{0}^{*}$, rather than partial correlations. We coin the resulting algorithm the ‘nonPC’ algorithm, to emphasize that it is a nonparametric generalization of parametric PC-stable algorithms.

The *oracle version* of the first step of nonPC, or the skeleton estimation step, is exactly the same as that of the PC-stable algorithm (Algorithm A1 in Appendix A). The second step, which extends the skeleton estimated in the first step to a CPDAG (Algorithm A2 in Appendix A), is comprised of some purely deterministic rules for edge orientations, and is exactly the same for both the nonPC and PC-stable as well. The only difference lies in the implementation of the tests for conditional independence relationships in the *sample versions* of the first step. Specifically, we replace all the conditional independence queries in the first step by tests based on $\rho_{0}^{*}\;\left(X,Y|Z\right)$. At some pre-specified significance level α, we infer that $X_{a}⫫X_{b}\;|\;X_{S}$ when ${\hat{\rho }}^{\;*}\left(X_{a},X_{b}|X_{S}\right)\leq \xi_{n,\alpha }$, where a,b∈V and S⊆V, |S|≠ϕ. When |S|=ϕ, ${\hat{\rho }}^{\;*}\left(X_{a},X_{b}|X_{S}\right)={\mathrm{dCov}}_{n}^{2}\left(X_{a},X_{b}\right)$ and $\rho_{0}^{*}\;\left(X,Y|Z\right)={\mathrm{dCov}}^{2}\left(X,Y\right)$. The critical value $\xi_{n,\alpha }$ in this case is obtained by a bootstrap procedure (see, e.g., Section 4 in [22] with d=2).

Given that the equivalence between conditional independence and zero partial correlations only holds for multivariate normal random variables, our generalization broadens the scope of applicability of causal structure learning by the PC/PC-stable algorithm to general distributions over DAGs. This nonparametric approach is thus a natural extension of Gaussian and Gaussian copula models. It enables capturing nonlinear and non-monotone conditional dependence relationships among the variables, which partial correlations fail to detect.

Next, we establish theoretical guarantees on the correctness of the nonPC algorithm in learning the true underlying causal structure in sparse high-dimensional settings. Our consistency results only require mild moment and tail conditions on the set of variables, without making any strict distributional assumptions. Denote by $m_{p}$ the maximum cardinality of the conditioning sets considered in the adjacency search step of the PC-stable algorithm. Clearly, $m_{p}\leq q$, where $q:=\max_{1\leq a\leq p}\;\left|\mathrm{adj}\left(G,a\right)\;\right|$ is the maximum degree of the DAG G. For a fixed pair of nodes a,b∈V, the conditioning sets considered in the adjacency search step are elements of $J_{a,b}^{m_{p}}:=\{S\subseteq V\setminus \left\{a,b\right\}:|S|\leq m_{p}\}$.

We first establish a concentration inequality that gives the rate at which the absolute difference of $\rho_{0}^{*}\;\left(X_{a},X_{b}|X_{S}\right)$ and its plug-in estimate ${\hat{\rho }}^{\;*}\left(X_{a},X_{b}|X_{S}\right)$ decays to zero, for any fixed pair of nodes *a* and b∈V and a fixed conditioning set *S*. Towards that, we impose the following regularity conditions.

(A1) There exists $s_{0}>0$ such that, for $0\leq s<s_{0}$, $\underset{p}{\sup}\max_{1\leq a\leq p}E\;\exp\left(sX_{a}^{2}\right)<\infty$.
(A2) The kernel function K(·) is non-negative and uniformly bounded over its support.

Condition (A1) imposes a sub-exponential tail bound on the squares of the random variables. This is a quite commonly used condition, for example, in the high-dimensional feature screening literature (see, for example, [23]). Condition (A2) is a mild condition on the kernel function K(·) that is guaranteed by many commonly used kernels, including the Gaussian kernel. Under conditions (A1) and (A2), the next result shows that the plug-in estimate ${\hat{\rho }}^{\;*}\left(X_{a},X_{b}|X_{S}\right)$ converges in probability to its population counterpart $\rho_{0}^{*}\;\left(X_{a},X_{b}|X_{S}\right)$ exponentially fast.

**Theorem** **1.** *Under conditions (A1) and (A2), for any  ϵ>0, there exist positive constants A, B and  γ∈(0,1/4)  such that*

$$\begin{array}{c} P\left(|\;{\hat{\rho }}^{\;*}\left(X_{a},X_{b}|X_{S}\right)-\rho_{0}^{*}\;\left(X_{a},X_{b}|X_{S}\right)\;|>\epsilon \right)\;\leq \;O\left(2\;\exp\left(-A\;n^{1-2\gamma }\;\epsilon^{2}\right)\;+\;n^{4}\;\exp\left(-B\;n^{\gamma }\right)\right)\;. \end{array}$$

The proof of Theorem 1 is long and somewhat technical; it is thus relegated to Appendix B. Theorem 1 serves as the main building block towards establishing the consistency of the nonPC algorithm in sparse high-dimensional settings.

For notational convenience, henceforth, we denote $\rho_{0}^{*}\;\left(X_{a},X_{b}|X_{S}\right)$ and ${\hat{\rho }}^{\;*}\left(X_{a},X_{b}|X_{S}\right)$ by $\rho_{0\;;\;a\;b|S}^{*}$ and ${\hat{\rho }}_{ab|S}^{\;*}$, respectively. In Theorem 2 below, we establish a uniform bound for the errors in inferring conditional independence relationships using the $\rho_{0}^{*}$-based test in the skeleton estimation step of the sample version of the nonPC algorithm.

**Theorem** **2.** *Under conditions (A1) and (A2), for any  ϵ>0, there exist positive constants A, B and  γ∈(0,1/4)  such that*

(7) $$\begin{array}{cc} \underset{\begin{array}{c} a,b\in V\\ S\in J_{a,b}^{m_{p}} \end{array}}{\sup}P\left(|\;{\hat{\rho }}_{ab|S}^{\;*}-\rho_{0\;;\;ab|S}^{*}\;|>\epsilon \right)\; & \leq \;P(\underset{\begin{array}{c} a,b\in V\\ S\in J_{a,b}^{m_{p}} \end{array}}{\sup}\left|\;{\hat{\rho }}_{ab|S}^{\;*}-\rho_{0\;;\;ab|S}^{*}\;\right|>\epsilon )\\ \; & \leq \;O\left(p^{m_{p}+2}\;\left[\;2\;\exp\left(-A\;n^{1-2\gamma }\;\epsilon^{2}\right)\;+\;n^{4}\;\exp\left(-B\;n^{\gamma }\right)\right]\right)\;. \end{array}$$

Next, we turn to proving the consistency of the nonPC algorithm in the high-dimensional setting where the dimension *p* can be much larger than the sample size *n*, but the DAG is considered to be sparse. We impose the following regularity conditions, which are similar to the assumptions imposed in Section 3.1 of [8] in order to prove the consistency of the PC algorithm for Gaussian graphical models. We let the number of variables *p* grow with the sample size *n* and consider $p=p_{n}$, and also the DAG $G=G_{n}:=\left(V_{n},E_{n}\right)$ and the distribution $P=P_{n}$.

(A3) The dimension $p_{n}$ grows at a rate such that the right-hand side of (7) tends to zero as n→∞. In particular, this is satisfied when $p_{n}=O\left(n^{r}\right)$ for any 0≤r<∞.
(A4) The maximum degree of the DAG $G_{n}$, denoted by $q_{n}:=\max_{1\leq a\leq p_{n}}\;\left|adj\left(G_{n},a\right)\;\right|$, grows at the rate of $O(n^{1-b})$, where 0<b≤1.
(A5) The distribution $P_{n}$ is faithful to the DAG  $G_{n}$  for all *n*. In other words, for any $a,b\in V_{n}$ and $S\in J_{a,b}^{m_{p_{n}}},$
  $$\begin{array}{c} X_{a}\;\;\mathrm{and}\;\;X_{b}\;\;\;\mathrm{are}d-\mathrm{separated}\mathrm{by}\;\;X_{S}\;\;⟺\;\;X_{a}⫫X_{b}\;|\;X_{S}\;\;⟺\;\;\rho_{0\;;\;a\;b|S}^{*}=0\;. \end{array}$$
  Moreover, $\rho_{0\;;\;a\;b|S}^{*}$ values are uniformly bounded both from above and below. Formally,
  $$\begin{array}{cc} C_{min}\;: & =\;\underset{\begin{array}{c} a,b\in V_{n}\\ S\in J_{a,b}^{m_{p_{n}}}\\ \rho_{0\;;\;ab|S}^{*}\neq 0 \end{array}}{\inf}\;\rho_{0\;;\;ab|S}^{*}\;\geq \;\lambda_{min}\;\;\lambda_{min}^{-1}=O\left(n^{v}\right)\\ \mathrm{and}\;C_{max}\;: & =\;\underset{\begin{array}{c} a,b\in V_{n}\\ S\in J_{a,b}^{m_{p_{n}}} \end{array}}{\sup}\;\rho_{0\;;\;ab|S}^{*}\;\leq \;\lambda_{max}\; \end{array}$$
  where $\lambda_{max}$ is a positive constant and 0<v<1/4.

Condition (A3) allows the dimension to grow at any arbitrary polynomial rate of the sample size. Condition (A4) is a sparsity assumption on the underlying true DAG, allowing the maximum degree of the DAG to also grow, but at a slower rate than *n*. Since $m_{p}\leq q_{n}$, we also have $m_{p}=O\left(n^{1-b}\right)$. Finally, Condition (A5) is the strong faithfulness assumption (Definition 1.3 in [24]) on $P_{n}$ and is similar to condition (A4) in [8]. This essentially requires $\rho_{0\;;\;ab|S}^{*}$ to be bounded away from zero when the vertices $X_{a}$ and $X_{b}$ are not d-separated by $X_{S}$. It is worth noting that the faithfulness assumption alone is not enough to prove the consistency of the PC/PC-stable/nonPC algorithms in high-dimensional settings, and the more stringent strong faithfulness condition is required.

**Remark** **2.** *For notational convenience, treat $X_{a},X_{b}$ and $X_{S}$ as X, Y and Z, respectively, for any $a,b\in V_{n}$ and $S\in J_{a,b}^{m_{p_{n}}}$. From Equation (3), we have*

$$\begin{array}{c} {\mathrm{CdCov}}^{2}\left(X,Y|Z\right)\;=\;\frac{1}{12}\;E\;\left[\;d_{1234}^{S}\;|\;Z_{1}=Z,\cdots ,Z_{4}=Z\right], \end{array}$$

*which implies*

$$\begin{array}{c} \rho_{0}^{*}\;=\;E\;\left[{\mathrm{CdCov}}^{2}\left(X,Y|Z\right)\right]\;=\;\frac{1}{12}\;E\;\left[\;d_{1234}^{S}\right]\;=\;\frac{1}{12}\;E\;\left[\;d_{1234}+d_{1243}+d_{1432}\right]\;. \end{array}$$

Condition (A1) implies  $\underset{p}{\sup}\max_{1\leq a\leq p}E\;X_{a}^{2}<\infty$. With this and the definition of $d_{ijkl}$ in Section 2.3, it follows from some simple algebra and the Cauchy–Schwarz inequality that  $\rho_{0}^{*}<\infty$. This provides a justification for the second part of Assumption (A5) that $\underset{\begin{array}{c} a,b\in V_{n}\\ S\in J_{a,b}^{m_{p_{n}}} \end{array}}{\sup}\;\rho_{0\;;\;ab|S}^{*}\;\leq \;\lambda_{max}$  for some positive constant $\lambda_{max}$.

The next theorem establishes that the nonPC algorithm consistently estimates the skeleton of a sparse high-dimensional DAG, thereby providing the necessary theoretical guarantees to our proposed methodology. It is worth noting that, in the sample version of the PC-stable and hence the nonPC algorithm, all the inference is done during the skeleton estimation step. The second step that involves appropriately orienting the edges of the estimated skeleton is purely deterministic (see Sections 4.2 and 4.3 in [7]). Therefore, to prove the consistency of the nonPC algorithm in estimating the equivalence class of the underlying true DAG, it is enough to prove the consistency of the estimated skeleton. We include the detailed proof of Theorem 3 in Appendix B.

**Theorem** **3.** *Assume that Conditions (A1)–(A5) hold. Let $G_{\mathrm{skel},n}$ be the true skeleton of the graph $G_{n}$, and ${\hat{G}}_{\mathrm{skel},n}$ be the skeleton estimated by the nonPC algorithm. Then, as n→∞, $P\left({\hat{G}}_{\mathrm{skel},n}=G_{\mathrm{skel},n}\right)\to 1$.*

**Remark** **3.** *In the proof of Theorem 3, we consider the threshold $\xi_{\alpha }$ to be of constant order. However, the proof continues to work as long as $\xi_{\alpha }$ is of the same order as $C_{min}$ as n→∞.*

### 3.2. The Nonparametric FCI Algorithm in High Dimensions

The FCI is a modification of the PC algorithm that accounts for latent and selection variables. Thus, generalizations of the PC algorithm naturally extend to the FCI as well. Similar to nonPC, we propose testing for conditional independence relations nonparametrically in the *sample version* of the FCI-stable algorithm (Algorithm A3 in Appendix A) based on $\rho_{0}^{*}$, instead of partial correlations. We coin the resulting algorithm the ‘nonFCI’ algorithm, to emphasize that it is a generalization of parametric FCI-stable algorithms. Again, the *oracle version* of the nonFCI is exactly the same as that of the FCI-stable algorithm. The difference is in the implementation of the tests for conditional independence relationships in their *sample versions*. This broadens the scope of the FCI algorithm in causal structural learning for observational data in the presence of latent and selection variables when Gaussianity is not a viable assumption. More specifically, it enables capturing nonlinear and non-monotone conditional dependence relationships among the variables that partial correlations would fail to detect.

Equipped with the theoretical guarantees we established for the nonPC in Section 3.1, we establish below in Theorem 4 the consistency of the nonFCI algorithm for general distributions in sparse high-dimensional settings. Let H=(V,E) be a DAG with the vertex set partitioned as $V=V_{X}\cup V_{L}\cup V_{T}$, where $V_{X}$ indexes the set of *p* observed variables, $V_{L}$ denotes the set of latent variables and $V_{T}$ stands for the set of selection variables. Let M be the unique MAG over $V_{X}$. We let *p* grow with *n* and consider $p=p_{n}$, $H=H_{n}$ and $Q=Q_{n}$, where *Q* is the distribution of $\left(U_{1},\cdots ,U_{p}\right):=(X_{1}\;|\;V_{T},\cdots ,X_{p}\;\left|\;V_{T}\right)$. We provide below the definition of possible-D-SEP sets (Definition 3.3 in [4]).

**Definition** **1.** *Let C be a graph with any of the following edge types: ∘−∘, ∘→ and ↔. A possible-D-SEP $\left(X_{a},X_{b}\right)$ in C, denoted $\mathrm{pds}(C,X_{a},X_{b})$, is defined as follows: $X_{c}\in$$\mathrm{pds}(C,X_{a},X_{b})$ if and only if there is a path π between $X_{a}$ and $X_{c}$ in C such that, for every subpath $\langle X_{e},X_{f},X_{g}\rangle$ of π, $X_{f}$ is a collider on the subpath in C or $\langle X_{e},X_{f},X_{g}\rangle$ is a triangle in C.*

To prove the consistency of the nonFCI algorithm in sparse high-dimensional settings, we impose the following regularity conditions, which are similar to the assumptions imposed in Section 4 in [4].

(C3) The distribution  $Q_{n}$ is faithful to the underlying MAG $M_{n}$ for all *n*.
(C4) The maximum size of the possible-D-SEP sets for finding the final skeleton in the FCI-stable algorithm (Algorithm A6 in Appendix A), $q_{n}^{\prime }$, grows at the rate of $O(n^{1-b})$, where 0<b≤1.
(C5) For any $U_{i},U_{j}\in \left\{U_{1},\cdots ,U_{p_{n}}\right\}$ and $U_{S}\subseteq \left\{U_{1},\cdots ,U_{p_{n}}\right\}\setminus \left\{U_{i},U_{j}\right\}$ with $|U_{S}|\leq q_{n}^{\prime }$, assume
  $$\begin{array}{cc} \; & \inf\;\left\{|\rho_{0}^{*}\left(U_{i},U_{j}|U_{S}\right)|:\rho_{0}^{*}\left(U_{i},U_{j}|U_{S}\right)\neq 0\right\}\;\geq \;\lambda_{min}^{\prime }\;\;{\left(\lambda_{min}^{\prime }\right)}^{-1}=O\left(n^{v}\right)\\ \mathrm{and}\; & \sup\;|\rho_{0}^{*}\left(U_{i},U_{j}|U_{S}\right)|\;\leq \;\lambda_{max}^{\prime }\; \end{array}$$
  where $\lambda_{max}^{\prime }$ is a positive constant  and   0<v<1/4.

**Theorem** **4.** Suppose conditions (A1)–(A3) and (C3)–(C5) hold. Denote by $C_{n}$ and $C_{n}^{*}$ the true underlying FCI-PAG and the output of the nonFCI algorithm, respectively. Then, as n→∞, $P\left(C_{n}^{*}=C_{n}\right)\to 1$.

## 4. Numerical Studies

### 4.1. Performance of the NonPC Algorithm

In this subsection, we compare the performances of the nonPC and the PC-stable algorithms in finding the skeleton and the CPDAG for various simulated datasets. We simulate random DAGs in the following examples and sample from probability distributions faithful to them.

**Example** **1** (Linear SEM). *We first fix a sparsity parameter s∈(0,1) and enumerate the vertices as V={1,⋯,p}. We then construct a p×p adjacency matrix *Λ* as follows. First, initialize *Λ* as a zero matrix. Next, fill every entry in the lower triangle (below the diagonal) of *Λ* by independent realizations of Bernoulli random variables with success probability s. Finally, replace each nonzero entry in *Λ* by independent realizations of a Uniform(0.1,1) random variable.*

In this scheme, each node has the same expected degree E(m)=(p−1)s, where *m* is the degree of a node and follows a Binomial (p−1,s) distribution. Using the adjacency matrix Λ, the data are then generated from the following linear structural equation model (SEM):

X=ΛX+ϵ

where $\epsilon =(\epsilon_{1},\cdots ,\epsilon_{p})$ and $\epsilon_{1},\cdots ,\epsilon_{p}$ are jointly independent. To obtain samples ${\left\{X_{1}^{k},\cdots ,X_{p}^{k}\right\}}_{k=1}^{n}$ on $\left\{X_{1},\cdots ,X_{p}\right\}$, we first sample ${\left\{\epsilon_{1}^{k},\cdots ,\epsilon_{p}^{k}\right\}}_{k=1}^{n}$ from the three following data-generating schemes. For 1≤k≤n and 1≤i≤p,

1. Normal: Generate $\epsilon_{i}^{k}$ ’s independently from a standard normal distribution.
2. Copula: Generate $\epsilon_{i}^{k}$ ’s as in (1) and then transform the marginals to a $F_{1,1}$ distribution.
3. Mixture: Generate $\epsilon_{i}^{k}$ ’s independently from a 50–50 mixture of a standard normal and a standard Cauchy distribution.

**Example** **2** (Nonlinear SEM). *In this example, we first generate a p×p adjacency matrix *Λ* in the similar way as in Example 1 and then generate the data from the following nonlinear SEM (similar to [10]): $X_{i}=\sum_{j\;:\;\Lambda_{ij}\neq 0}f_{ij}\left(X_{j}\right)+\epsilon_{i}$  with  $\epsilon_{i}\overset{i.i.d.}{∼}N\left(0,1\right)$, where 1≤j<i≤p. If the functions $f_{ij}$’s are chosen to be nonlinear, then the data will typically not correspond to a well-known multivariate distribution. We consider $f_{ij}\left(x_{j}\right)=b_{ij1}x_{j}+b_{ij2}x_{j}^{2}$, where $b_{ij1}$ and $b_{ij1}$ are independently sampled from N(0,1) and N(0,0.5) distributions, respectively.*

With the exception of Example 1.1, the above examples are all non-Gaussian graphical models. We would thus expect the nonPC to perform better than the PC-stable in learning the unknown causal structure in these examples. For each of the four data generating methods considered above, we compare the Structural Hamming Distance (SHD) [25] between the estimated and the true skeletons of the underlying DAGs using the nonPC and PC-stable algorithms. The SHD between two undirected graphs is the number of edge additions or deletions necessary to make the two graphs match. Therefore, larger SHD values between the estimated and the true skeleton correspond to worse estimates.

We consider 199 bootstrap replicates for the CdCov-based conditional independence tests in the implementation of our nonPC algorithm and the significance level α=0.05. Table 1 presents the average SHD for the different data generating schemes over 20 simulation runs, for different choices of n,p and E(m).

The results in Table 1 demonstrate that the nonPC performs nearly as good as the PC-stable for the Gaussian data example, in terms of the average SHD. However, for each of the non-Gaussian data examples, the nonPC performs better than the PC-stable in estimating the true skeleton of the underlying DAGs. The improvement in SHD becomes more substantial as the dimension grows. The superior performance of the nonPC over PC-stable for the non-Gaussian graphical models is expected, as the characterization of conditional independence by partial correlations is only valid under the assumption of joint Gaussianity.

### 4.2. Performance of the NonFCI Algorithm

In this subsection, we compare the performances of the nonFCI and the FCI-stable algorithms over various simulated datasets. We first generate random DAGs as in Examples 1 and 2. To assess the impact of latent variables, we randomly define half of the variables with no parents and at least one child as latent. We do not consider selection variables. We run both the nonFCI and the FCI-stable algorithms on the above data examples with n=200, p={10,20,30,100,200} and α=0.01, using 199 bootstrap replicates for the CdCov-based conditional independence tests. We consider 20 simulation runs for each of the data generating models. Table 2 reports the average SHD between the estimated and true PAG skeleton by the nonFCI and FCI-stable algorithms.

The results in Table 2 demonstrate that, in both the Gaussian and non-Gaussian examples, the nonFCI algorithm outperforms the FCI-stable in estimating the true PAG skeleton.

### 4.3. Real Data Example

A major difficulty in assessing whether nonPC and nonFCI provide more reasonable estimates compared to the parametric versions of the algorithms in high-dimensional real data settings is that the true causal graph is not known in most of the cases. In absence of the truth, we may only be able to draw some conclusions about sensible causal mechanisms by examining known or logical relationships among pairs of variables. However, this becomes increasingly difficult for larger networks, where even visualization becomes challenging. This is why we first choose a relatively smaller dataset in Section 4.3.1, where we can draw upon background knowledge to glean insight into potential causal mechanisms in a setting where the data are clearly non-Gaussian. This example highlights the main focus of the paper that, with non-Gaussian data (categorical, as in this example), nonPC is expected to perform better than the PC-stable in learning the true causal structure of the underlying DAG. In Section 4.3.2, we consider a larger example and examine the performance of PC-stable and nonPC in learning the DAG from both seemingly Gaussian data as well as a categorized version of the same data. This example clearly illustrates the potential limitations of PC-stable: in contrast to nonPC, the output of PC-stable can be strikingly different when applied to a categorized version of the original data.

#### 4.3.1. Montana Poll Dataset

To demonstrate the flexibility of our proposed framework, we first apply the nonPC algorithm to the Montana Economic Outlook Poll dataset. The poll was conducted in May 1992 where a random sample of 209 Montana residents were asked whether their personal financial status was worse, the same or better than a year ago, and whether they thought the state economic outlook was better than the year before. Accompanying demographic information on the respondents’ age, income, political orientation, and area of residence in the state were also recorded. We obtained the dataset from the Data and Story Library (DASL), available at https://math.tntech.edu/e-stat/DASL/page4.html (accessed on 25 March 2021). The study is comprised of the following seven categorical variables: AGE = 1 for under 35, 2  for 35–54, 3  for 55 and over;  SEX = 0   for male, 1  for female;  INC = yearly income: 1 for under $20 K, 2  for $20–35 K, 3  for over $35 K;  POL = 1 for Democrat, 2  for Independent, 3  for Republican;  AREA = 1 for Western, 2  for Northeastern, 3  for Southeastern Montana;  FIN (=Financial status): 1 for worse, 2  for same, 3  for better than a year ago;  and  STAT (=State economic outlook): 1 for better, 0  for not better than a year ago.

**Figure 1:**
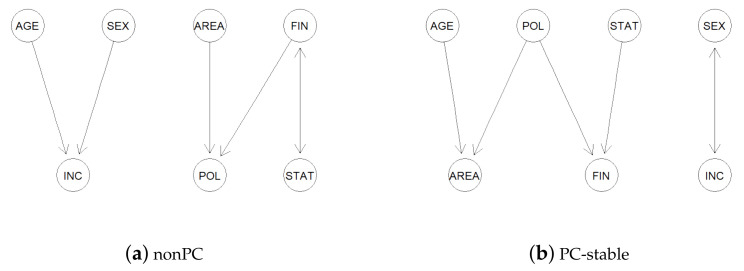
CPDAGs estimated by the nonPC and PC-stable algorithms for the Montana poll dataset.

After removing the cases with missing values, we are left with n=163 samples. Since all the variables are categorical, the Gaussianity assumption is outrightly violated. Thus, we would expect the nonPC to perform better than the PC-stable in learning the true causal structure among the variables in this case. Figure 1 below presents the CPDAGs estimated by the nonPC and PC-stable algorithms at a significance level α=0.1. We consider 199 bootstrap replicates for the CdCov-based conditional independence tests in the implementation of the nonPC algorithm.

It is quite intuitive that age and sex are likely to affect the income; one’s financial status and the area of residence might also influence their political inclination; and improvements or downturns in the state economic outlook might impact an individual’s financial status. The CPDAG estimated by the nonPC algorithm in Figure 1a affirms such common-sense understanding of these causal influences. However, in the CPDAG estimated by the PC-stable in Figure 1b, the edge between age and income is missing. In addition, the directed edges POL → AREA and POL → FIN seem to make little sense in this case.

#### 4.3.2. Protein Expression Data

We next consider a protein expression dataset of 410 patients with breast cancer from The Cancer Genome Atlas (TCGA). The dataset consists of p=118 genes, and we randomly select a subset of n=100 patients with PR-negative status. Since the true causal structure of the genes in the cancer cells may be different than that of normal cells [26], we apply both the nonPC and PC-stable algorithms to learn the causal structure. To put the performances of the nonPC and PC-stable under scrutiny as the data depart farther away from Gaussianity, we categorize the protein expression data for each of the *p* genes, denoted by ${\left\{X_{a}^{k}\right\}}_{k=1}^{n}$, 1≤a≤p, as follows. We compute the three quartiles $Q_{1\;;\;a},\;Q_{2\;;\;a}$ and $Q_{3\;;\;a}$ of the protein expression values for every 1≤a≤p. Consequently, we obtain categorized protein expressions ${\left\{X_{C\;;\;a}^{k}\right\}}_{k=1}^{n}$ for 1≤a≤p, where

$$\begin{array}{c} X_{C\;;\;a}^{k}\;:=\;\left\{\begin{array}{cc} 0\; & \;\mathrm{if}\;X_{a}^{k}\leq Q_{1\;;\;a}\;\\ 1\; & \;\mathrm{if}\;Q_{1\;;\;a}<X_{a}^{k}\leq Q_{2\;;\;a}\;\\ 2\; & \;\mathrm{if}\;Q_{2\;;\;a}<X_{a}^{k}\leq Q_{3\;;\;a}\;\\ 3\; & \;\mathrm{if}\;X_{a}^{k}>Q_{3\;;\;a}\;. \end{array}\right. \end{array}$$

We apply the nonPC and PC-stable algorithms to both the original and the categorized protein expression data at a significance level α=0.01. We consider 199 bootstrap replicates for the CdCov-based conditional independence tests in the implementation of the nonPC algorithm. Table 3 below shows the SHD between the skeletons estimated from the original and the categorized data by the nonPC and PC-stable algorithms. It can be seen that the SHD between the skeletons estimated from the original and categorized data by the PC-stable algorithm is much larger than that for nonPC. This example highlights the potential limitation of parametric implementations of the PC algorithm: when the data deviate farther away from Gaussianity (in this case, being categorical), the estimates produced by the PC-stable may deviate considerably more from the estimates from the original data. In contrast, the nonparametric test in nonPC delivers more stable estimates regardless of the data distribution.

## 5. Discussion

We proposed nonparametric variants of the widely popular PC-stable and FCI-stable algorithms, which employ conditional distance covariance (CdCov) to test for conditional independence relationships in their sample versions. Our proposed algorithms broaden the applicability of the PC/PC-stable and FCI/FCI-stable algorithms to general distributions over DAGs, and enable taking into account nonlinear and non-monotone conditional dependence among the random variables, which partial correlations fail to capture. We show that the high-dimensional consistency of the PC-stable and FCI-stable algorithms carry over to more general distributions over DAGs when we implement CdCov-based nonparametric tests for conditional independence. These results are obtained without imposing any strict distributional assumptions and only require moment and tail conditions on the variables.

There are several intriguing potential directions for future research. First, it is generally difficult to select the tuning parameter (i.e., the significance threshold for the CdCov test) in causal structure learning. One possible strategy is to use ideas based on *stability selection* [27,28]. By assessing the stability of the estimated graphs in multiple subsamples, this strategy allows us to choose the tuning parameter in order to control the false positive error. However, the repeated subsampling increases the computational burden. Second, the computational and sample complexities of the PC and FCI algorithms (and hence those of the nonPC and nonFCI) scale with the maximum degree of the DAG, which is assumed to be small relative to the sample size. However, in many applications, one encounters sparse graphs containing a small number of highly connected ‘hub’ nodes. In such cases, ref. [29] proposed a low-complexity variant of the PC algorithm, namely the *reduced PC* (rPC) algorithm that exploits the local separation property of large random networks [30]. The rPC is shown to consistently estimate the skeleton of a high-dimensional DAG by conditioning only on sets of small cardinality. More recently, ref. [31] have generalized this approach to account for unobserved confounders. In this light, it would be intriguing to develop computationally faster variants of the nonPC and nonFCI in the future by exploiting the idea of local separation.

## Figures and Tables

**Table 1:** Comparison of the average structural Hamming distances (SHD) of nonPC and PC-stable algorithms across simulation studies.

			Normal		Copula	
*n*	*p*	E(m)	nonPC	PC-stable	nonPC	PC-stable
50	9	1.4	3.35	3.05	5.55	5.75
100	27	2.0	14.55	11.00	25.6	28.6
150	81	2.4	53.70	43.45	97.3	121.3
200	243	2.8	186.2	183.4	331.00	471.45
			Mixture		Nonlinear SEM	
*n*	*p*	E(m)	nonPC	PC-stable	nonPC	PC-stable
50	9	1.4	3.8	3.5	2.9	3.7
100	27	2.0	17.75	18.00	15.05	20.05
150	81	2.4	69.05	77.75	62.583	95.083
200	243	2.8	250.3	336.1	213.70	375.45

**Table 2:** Comparison of the average structural Hamming distances (SHD) of nonFCI and FCI-stable algorithms across simulation studies.

		Normal		Copula		Mixture		Nonlinear SEM	
*p*	E(m)	nonFCI	FCI-Stable	nonFCI	FCI-Stable	nonFCI	FCI-Stable	nonFCI	FCI-Stable
10	2.0	7.15	7.60	1.3	1.8	5.65	6.80	7.15	8.20
20	2.0	14.55	17.60	4.55	6.85	13.65	18.55	19.0	20.8
30	2.0	27.65	33.95	5.25	10.15	19.3	27.8	33.40	37.85
100	3.0	109.30	150.35	26.95	60.05	62.25	111.10	115.2	149.0
200	3.0	287.75	371.40	76.733	157.267	136.05	255.10	289.6	354.1

**Table 3:** Comparison of the SHD between the skeletons estimated from the original and the categorized protein expression data by the nonPC and PC-stable algorithms.

nonPC	PC-Stable
22	79

## Data Availability

The Montana Poll dataset has been accessed from the Data and Story Library (DASL) at https://math.tntech.edu/e-stat/DASL/page4.html (accessed on 25 March 2021).

## Appendix A. Preliminaries and Background

For the sake of completeness, we illustrate in this section the pseudocodes of the oracle versions of the PC-stable and FCI-stable algorithms. We also outline a local bootstrap procedure that can be used to approximate the threshold $\xi_{\alpha }$ mentioned in Section 3.1 and is used throughout the numerical studies in the paper.

Algorithm A1 presents the pseudocode of the oracle version of Step 1 of the PC-stable algorithm (Algorithm 4.1 of [7]), which estimates the skeleton of the underlying DAG. Algorithm A2 presents the pseudocode of Step 2 of the PC-stable algorithm (Algorithm 2 of [8]) that extends the skeleton estimated in Step 1 to the CPDAG. Algorithm A3 presents the pseudocode of the FCI-stable algorithm (Section 4.4 in [7]). It implements Algorithm A4 to obtain an initial skeleton of the underlying PAG, Algorithm A5 to orient the v-structures, and finally Algorithm A6 to obtain the final skeleton that the FCI-stable returns.

To approximate the threshold $\xi_{\alpha }$ to test for $H_{0}:X⫫Y|Z$  vs. HA:X⫫Y|Z at level α∈(0,1) (see Section 3.1), we consider the following local bootstrap procedure in the light of Section 4.3 in [15]. Given the i.i.d. sample ${\left\{W_{i}=\left(X_{i},Y_{i},Z_{i}\right)\right\}}_{i=1}^{n}$ from the joint distribution of W=(X,Y,Z), draw a local bootstrap sample ${\left\{W_{i}^{†}=\left(X_{i}^{†},Y_{i},Z_{i}\right)\right\}}_{i=1}^{n}$ and compute the bootstrap statistic. The detailed steps are as follows:

**Algorithm 1** Step 1 of the PC-stable algorithm (oracle version).
**Require** : Conditional independence information among all variables in *V*, and an ordering order(V) on the variables. Form the complete undirected graph C on the vertex set *V*. Let l=−1; **repeat** l=l+1; **for** all vertices $X_{a}$ in C **do** let $u\left(X_{a}\right)=adj\left(C,X_{a}\right)$ **end for** **repeat** Select a (new) ordered pair of vertices $\left(X_{a},X_{b}\right)$ that are adjacent in C such that $|u\left(X_{a}\right)\setminus \left\{X_{b}\right\}|\geq l$, using order (V); **repeat** Choose a (new) set $S\subseteq u\left(X_{a}\right)\setminus \left\{X_{b}\right\}$ with |S|=l, using order(V); **if** $X_{a}⫫X_{b}\;|\;S$ **then** Delete the edge $X_{a}-X_{b}$ from C; Let *sepset *$(X_{a},X_{b})=\;$*sepset *$(X_{b},X_{a})=S$; **end if** **until** $X_{a}$ and $X_{b}$ are no longer adjacent in C or all $S\subseteq u\left(X_{a}\right)\setminus \left\{X_{b}\right\}$ with |S|=l have been considered **until** all ordered pairs of adjacent vertices $\left(X_{a},X_{b}\right)$ in C with $|u\left(X_{a}\right)\setminus \left\{X_{b}\right\}|\geq l$ have been considered **until** all pairs of adjacent vertices $\left(X_{a},X_{b}\right)$ in C satisfy $|u\left(X_{a}\right)\setminus \left\{X_{b}\right\}|\leq l$ **Output** : The estimated skeleton C, separation sets *sepset*.

**Algorithm 2** Step 2 of the PC-stable algorithm.
**Require** : Skeleton C, separation sets *sepset*. **for** all all pair of nonadjacent vertices $X_{a},X_{c}$ with common neighbor $X_{b}$ in C **do** **if** $X_{b}\notin sepset\left(X_{a},X_{c}\right)$ **then** Replace $X_{a}-X_{b}-X_{c}$ in C by $X_{a}\to X_{b}\leftarrow X_{c}$; **end if** **end for** In the resulting PDAG, try to orient as many undirected edges as possible by repeated applications of the following rules : **(R1)** Orient $X_{b}-X_{c}$ into $X_{b}\to X_{c}$ whenever there is an arrow $X_{a}\to X_{b}$ such that $X_{a}$ and $X_{c}$ are nonadjacent (otherwise, a new v-structure is created). **(R2)** Orient $X_{a}-X_{c}$ into $X_{a}\to X_{c}$ whenever there is a chain $X_{a}\to X_{b}\to X_{c}$ (otherwise, a directed cycle is created). **(R3)** Orient $X_{a}-X_{c}$ into $X_{a}\to X_{c}$ whenever there are two chains $X_{a}-X_{b}\to X_{c}$ and $X_{a}-X_{d}\to X_{c}$ such that $X_{b}$ and $X_{d}$ are nonadjacent (otherwise, a new v-structure or a directed cycle is created).

**Algorithm A3** The FCI-stable algorithm (oracle version).
**Require** : Conditional independence information among all variables in $V_{X}$ given $V_{T}$. Use Algorithm A4 to find an initial skeleton (C), separation sets (sepset) and unshielded triple list (M); Use Algorithm A5 to orient v-structures (update C); Use Algorithm A6 to find the final skeleton (update C and sepset); Use Algorithm A5 to orient v-structures (update C); Use rules (R1)-(R10) of [6] to orient as many edge marks as possible (update C); **Output** : C, sepset.

**Algorithm A4** Obtaining an initial skeleton in the FCI-stable algorithm (Algorithm 4.1 in the supplement of [4]).
**Require** : Conditional independence information among all variables in $V_{X}$ given $V_{T}$, and an ordering order$\left(V_{X}\right)$ on the variables. Form the complete undirected graph C on the vertex set $V_{X}$ with edges ∘−∘. Let l=−1; **repeat** l=l+1; **for** all vertices $X_{a}$ in C **do** let $u\left(X_{a}\right)=adj\left(C,X_{a}\right)$ **end for** **repeat** Select a (new) ordered pair of vertices $\left(X_{a},X_{b}\right)$ that are adjacent in C such that $|u\left(X_{a}\right)\setminus \left\{X_{b}\right\}|\geq l$, using order $\left(V_{X}\right)$; **repeat** Choose a (new) set $Y\subseteq u\left(X_{a}\right)\setminus \left\{X_{b}\right\}$ with |Y|=l, using order$\left(V_{X}\right)$; **if** $X_{a}⫫X_{b}\;|\;Y\cup V_{T}$ **then** Delete the edge $X_{a}\circ \;-\;\circ X_{b}$ from C; Let sepset$(X_{a},X_{b})=\;$sepset$(X_{b},X_{a})=Y$; **end if** **until** $X_{a}$ and $X_{b}$ are no longer adjacent in C or all $Y\subseteq u\left(X_{a}\right)\setminus \left\{X_{b}\right\}$ with |Y|=l have been considered **until** all ordered pairs of adjacent vertices $\left(X_{a},X_{b}\right)$ in C with $|u\left(X_{a}\right)\setminus \left\{X_{b}\right\}|\geq l$ have been considered **until** all pairs of adjacent vertices $\left(X_{a},X_{b}\right)$ in C satisfy $|u\left(X_{a}\right)\setminus \left\{X_{b}\right\}|\leq l$ Form a list M of all unshielded triples $\langle X_{c}\;\cdot \;X_{d}\rangle$ (i.e., the middle vertex is left unspecified) in C with c<d. **Output** : C, sepset, M.

**Algorithm A5** Orienting v-structures in the FCI-stable algorithm (Algorithm 4.2 in the supplement of [4]).
**Require** : Initial skeleton (C), separation sets (sepset) and unshielded triple list (M). **for** all elements $\langle X_{a},X_{b},X_{c}\rangle$ of M **do** **if** $X_{b}\notin \mathrm{sepset}\left(X_{a},X_{c}\right)$ **then** Orient $X_{a}★\;-\;\circ X_{b}\circ \;-\;★X_{c}$ as $X_{a}★\;\to X_{b}\leftarrow \;★X_{c}$ **end if** **end for** **Output** : C, sepset.

**Algorithm A6** Obtaining the final skeleton in the FCI-stable algorithm (Algorithm 4.3 in the supplement of [4]).
**Require**: Partially oriented graph (C) and separation sets (sepset). **for** all vertices $X_{a}$ in C **do** let $v\left(X_{a}\right)=\mathrm{pds}\left(C,X_{a},\cdot \right)$; **for** all vertices $X_{b}\in adj\left(C,X_{a}\right)$ **do** Let l=−1; **repeat** l=l+1; **repeat** Choose a (new) set $Y\subseteq v\left(X_{a}\right)\setminus \left\{X_{b}\right\}$ with |Y|=l; **if** $X_{a}⫫X_{b}\;|\;Y\cup V_{T}$ **then** Delete the edge $X_{a}★\;-\;★X_{b}$ from C; Let sepset$(X_{a},X_{b})=\;$sepset$(X_{b},X_{a})=Y$; **end if** **until** $X_{a}$ and $X_{b}$ are no longer adjacent in C or all $Y\subseteq v\left(X_{a}\right)\setminus \left\{X_{b}\right\}$ with |Y|=l have been considered **until** $X_{a}$ and $X_{b}$ are no longer adjacent in C or $|v\left(X_{a}\right)\setminus \left\{X_{b}\right\}|<l$ **end for** **end for** Reorient all edges in C as ∘−∘. Form a list M of all unshielded triples $\langle X_{c}\;\cdot \;X_{d}\rangle$ in C with c<d. **Output** : C, sepset, M.

A. For i=1,⋯,n, draw $X_{i}^{†}$ from
  $$\begin{array}{c} {\hat{F}}_{X|Z=Z_{i}}\;=\;\frac{\sum_{j=1}^{n}K_{ij}\;1\left(-\infty ,X_{j}\right]\left(x\right)}{\sum_{j=1}^{n}K_{ij}}\;. \end{array}$$
  Compute ${\hat{\rho }}^{\;*†}$ based on the local bootstrap sample ${\left\{W_{i}^{†}=\left(X_{i}^{†},Y_{i},Z_{i}\right)\right\}}_{i=1}^{n}$.
B. Repeat Step A *B* times to obtain ${\left\{{\hat{\rho }}_{b}^{\;*†}\right\}}_{b=1}^{B}$. Obtain $\xi_{n,\alpha }^{\;*}$ as the $100{\left(1-\alpha \right)}^{th}$ percentile of ${\left\{nh^{r/2}\;{\hat{\rho }}_{b}^{\;*†}\right\}}_{b=1}^{B}$. Then, $\frac{1}{nh^{r/2}}\;\xi_{n,\alpha }^{\;*}$ can be considered as an approximation for $\xi_{\alpha }$.

## Appendix B. Proofs of the Theoretical Results

In this section, we provide detailed technical proofs of the theoretical results presented in the paper. We first state a concentration inequality in Lemma A1. The result in Lemma A1 is not new and can be seen as a corollary of Theorem A in Section 5.6.1 of [32]; however, it is a key technical ingredient in the proof of Theorem 1, which is the main theoretical innovation of our paper. For completeness, we include a short proof for Lemma A1.

**Lemma** **A1.**
*Consider a U-statistic Un=U(X1,⋯,Xn)=nm−1∑i1<⋯<imh(Xi1,⋯,Xim) with a symmetric kernel h such that $E\;U_{n}=E\;h\left(X_{1},\cdots ,X_{m}\right)=\theta$. Further suppose $|h(X_{1},\cdots ,X_{m})|\leq M$ for some M>0. Then, for any ϵ>0, we have*

$$\begin{array}{c} P\left(|U_{n}-\theta |>\epsilon \right)\;\leq \;2\;\exp\left(-\frac{\epsilon^{2}\;k}{2M^{2}}\right)\; \end{array}$$

*where $k:=\lfloor \frac{n}{m}\rfloor$.*

**Proof** **of** **Lemma** **A1.** Define

$$W\left(X_{1},\cdots ,X_{n}\right)\;:=\;\frac{1}{k}\;\left[h\left(X_{1},\cdots ,X_{m}\right)\;+\;h\left(X_{m+1},\cdots ,X_{2m}\right)\;+\;\cdots \;+\;h\left(X_{km-m+1},\cdots ,X_{km}\right)\;\right]\;.$$

Then, following Section 5.1.6 in [32], we can write

(A1) $$U_{n}\;=\;\frac{1}{n!}\sum_{\pi }W\left(X_{i_{1}},\cdots ,X_{i_{n}}\right)\;$$

where $\sum_{\pi }$ denotes summation over all n! permutations $\left(i_{1},\cdots ,i_{n}\right)$ of (1,2,⋯,n). Thus, $U_{n}$ can be expressed as an average of n! terms, each of which is an average of *k* i.i.d. random variables. Using Markov’s inequality, convexity of the exponential function and Jensen’s inequality, we have, for any t>0,

(A2) $$\begin{array}{c} \begin{array}{cc} P\left(U_{n}-\theta >\epsilon \right)\; & =\;P\left(\exp\left(t\;(U_{n}-\theta )\right)>\exp\left(t\epsilon \right)\right)\\ \; & \leq \;\exp\left(-t\epsilon \right)\exp\left(-t\theta \right)\;E\;\left[\exp\left(t\;U_{n}\right)\right]\\ \; & =\;\exp\left(-t\epsilon \right)\exp\left(-t\theta \right)\;E\left[\exp\left(t\;\frac{1}{n!}\sum_{p}W\left(X_{i_{1}},\cdots ,X_{i_{n}}\right)\right)\right]\\ \; & \leq \;\exp\left(-t\epsilon \right)\exp\left(-t\theta \right)\;\frac{1}{n!}\sum_{\pi }E\left[\exp\left(t\;W(X_{i_{1}},\cdots ,X_{i_{n}})\right)\right]\\ \; & =\;\exp\left(-t\epsilon \right)\exp\left(-t\theta \right)\;{\left[E\left(\exp\left(\frac{t}{k}\;h\right)\right)\right]}^{k}\\ \; & =\;\exp\left(-t\epsilon \right)\;E^{k}\left[\exp\left(\frac{t}{k}\left(h-\theta \right)\right)\right]\; \end{array} \end{array}$$

where, for notational simplicity, we use *h* to denote $h(X_{1},\cdots ,X_{m})$. Using Hoeffding’s Lemma, we have from (A2)

$$\begin{array}{c} P\left(U_{n}-\theta >\epsilon \right)\;\leq \;\exp\left(-t\epsilon \;+\;k\;\frac{1}{8}\;\frac{t^{2}}{k^{2}}\;{\left(2M\right)}^{2}\right)\;=\;\exp\left(-t\epsilon \;+\;\frac{t^{2}M^{2}}{2k}\right)\;. \end{array}$$

Symmetrically, we obtain

(A3) $$P\left(|U_{n}-\theta |>\epsilon \right)\;\leq \;2\;\exp\left(-t\epsilon \;+\;\frac{t^{2}M^{2}}{2k}\right)\;.$$

The right-hand side of (A3) is minimized at $t=\epsilon \;k/M^{2}$. Therefore, choosing $t=\epsilon \;k/M^{2}$, we obtain

$$\begin{array}{c} P\left(|U_{n}-\theta |>\epsilon \right)\;\leq \;2\;\exp\left(-\frac{\epsilon^{2}\;k}{2M^{2}}\right)\;. \end{array}$$

□

**Proof** **of** **Theorem** **1.** When |S|=0, it can be shown in similar lines of Theorem 1 in Li et al. (2012) [33] that, for any ϵ>0, there exist positive constants *A*, *B* and γ∈(0,1/4) such that

$$\begin{array}{c} P\left(|\;{\hat{\rho }}^{\;*}\left(X_{a},X_{b}|X_{S}\right)-\rho_{0}^{*}\;\left(X_{a},X_{b}|X_{S}\right)\;|>\epsilon \right)\;\leq \;O\left(2\;\exp\left(-A\;n^{1-2\gamma }\;\epsilon^{2}\right)\;+\;n\;\exp\left(-B\;n^{\gamma }\right)\right)\;. \end{array}$$

Now, consider the case $0<|S|\leq m_{p}$. For notational convenience, we treat $X_{a},X_{b}$ and $X_{S}$ as *X*, *Y* and *Z*, respectively. Denote $\delta_{Z}:={\mathrm{CdCov}}^{2}\left(X,Y|Z\right)$. Then, $\rho_{0}^{*}=E\left[\delta_{Z}\right]$. Recall that

(A4) $$\begin{array}{c} \begin{array}{cc} \; & {\hat{\rho }}^{\;*}\left(X,Y|Z\right)\;:=\;\frac{1}{n}\sum_{u=1}^{n}{\mathrm{CdCov}}_{n}^{2}\left(X,Y|Z_{u}\right)\;\;:=\;\frac{1}{n}\sum_{u=1}^{n}\Delta_{i,j,k,l;u}\;\\ \mathrm{where}\; & \Delta_{i,j,k,l;u}\;:=\;\sum_{i,j,k,l}\frac{K_{iu}\;K_{ju}\;K_{ku}\;K_{lu}}{12\;{\left(\sum_{i=1}^{n}K_{iu}\right)}^{4}}\;d_{ijkl}^{S}\;. \end{array} \end{array}$$

From (A4), we have

(A5) $$\begin{array}{c} \begin{array}{cc} \; & E\;\left[{\mathrm{CdCov}}_{n}^{2}\left(X,Y|Z_{u}\right)|Z\right]\\ \; & =\;\frac{1}{12}\;E\;\left[\;d_{1234}^{S}\;|\;Z_{1}=Z_{u},\cdots ,Z_{4}=Z_{u}\right]\;\sum_{i,j,k,l}K_{iu}\;K_{ju}\;K_{ku}\;K_{lu}\;/\;{\left(\sum_{i=1}^{n}K_{iu}\right)}^{4}\\ \; & =\;\frac{1}{12}\;E\;\left[\;d_{1234}^{S}\;|\;Z_{1}=Z_{u},\cdots ,Z_{4}=Z_{u}\right]\;=\;\delta_{Z_{u}}\; \end{array} \end{array}$$

where the last equality follows from Lemma 1 in [15]. Together, (A4) and (A5) imply $E\;\left[\;{\hat{\rho }}^{\;*}\right]\;=\;\rho_{0}^{*}$. Now, consider the truncation

(A6) $$\begin{array}{c} \begin{array}{cc} \rho_{0}^{*}\; & =\;\rho_{01}^{*}\;+\;\rho_{02}^{*}\\ \; & :=\;E\;\left[\frac{1}{12}\;d_{i,j,k,l}^{S}\;1\left(\left|\frac{1}{12}\;d_{i,j,k,l}^{S}\right|\leq M\right)\right]\;+\;E\;\left[\frac{1}{12}\;d_{i,j,k,l}^{S}\;1\left(\left|\frac{1}{12}\;d_{i,j,k,l}^{S}\right|>M\right)\right]\; \end{array} \end{array}$$

where M>0 will be specified later. Then, using triangle inequality,

(A7) $$\begin{array}{c} \begin{array}{cc} P\left(|{\hat{\rho }}^{\;*}-\rho_{0}^{*}|>\epsilon \right)\; & =\;P\left(\left|\frac{1}{n}\sum_{u=1}^{n}\left(\sum_{i,j,k,l}\Delta_{i,j,k,l;u}-\rho_{0}^{*}\right)\right|>\epsilon \right)\\ \; & \leq \;P\left(\left|\frac{1}{n}\sum_{u=1}^{n}\left(\sum_{i,j,k,l}\Delta_{i,j,k,l;u}\;1\left(\left|\frac{1}{12}\;d_{i,j,k,l}^{S}\right|\leq M\right)-\rho_{01}^{*}\right)\right|>\epsilon /2\right)\\ \; & \;\;+\;P\left(\left|\frac{1}{n}\sum_{u=1}^{n}\left(\sum_{i,j,k,l}\Delta_{i,j,k,l;u}\;1\left(\left|\frac{1}{12}\;d_{i,j,k,l}^{S}\right|>M\right)-\rho_{02}^{*}\right)\right|>\epsilon /2\right)\\ \; & =:I\;+\;\mathrm{II}\;. \end{array} \end{array}$$

Clearly, from (A4), we have $|\Delta_{i,j,k,l;u}|\leq M$ when $\left|\frac{1}{12}\;d_{i,j,k,l}^{S}\right|\leq M$. With this observation, we have

(A8) $$\begin{array}{cc} I\; & \leq \;2\;\exp\left(-\frac{n\;\epsilon^{2}}{8\;M^{2}}\right)\; \end{array}$$

which follows from Lemma A1 by setting m=1, k=⌊n⌋ and ϵ=ϵ/2. Choosing $M=c\;n^{\gamma }$ for γ∈(0,1/4) and some positive constant *c*, it follows from (A8) that

(A9) $$\begin{array}{cc} I\; & \leq \;2\;\exp\left(-C_{1}\;n^{1-2\gamma }\;\epsilon^{2}\right)\; \end{array}$$

for some $C_{1}>0$. Now, to find a suitable upper bound for II, note that a simple application of triangle inequality yields

(A10) $$\begin{array}{c} \begin{array}{cc} \frac{\epsilon }{2}\; & <\;\left|\;\frac{1}{n}\sum_{u=1}^{n}\sum_{i,j,k,l}\Delta_{i,j,k,l;u}\;1\left(\left|\frac{1}{12}\;d_{i,j,k,l}^{S}\right|>M\right)-\rho_{02}^{*}\;\right|\\ \; & \leq \;\left|\;\frac{1}{n}\sum_{u=1}^{n}\sum_{i,j,k,l}\Delta_{i,j,k,l;u}\;1\left(\left|\frac{1}{12}\;d_{i,j,k,l}^{S}\right|>M\right)\;\right|\;+\;\left|\rho_{02}^{*}\right|\;. \end{array} \end{array}$$

For the choice of $M=c\;n^{\gamma }$, we have

(A11) $$\begin{array}{c} \rho_{02}^{*}\;=\;E\;\left[\frac{1}{12}\;d_{i,j,k,l}^{S}\;1\left(\left|\frac{1}{12}\;d_{i,j,k,l}^{S}\right|>M\right)\right]\;<\;\frac{\epsilon }{4} \end{array}$$

for sufficiently large *n* (see, for example, Exercise 6 in Chapter 5, [34]). Combining (A10) and (A11), we obtain

$$\begin{array}{cc} \; & \left\{\left|\;\frac{1}{n}\sum_{u=1}^{n}\sum_{i,j,k,l}\Delta_{i,j,k,l;u}\;1\left(\left|\frac{1}{12}\;d_{i,j,k,l}^{S}\right|>M\right)-\rho_{02}^{*}\;\right|>\epsilon /2\right\}\\ \; & \subseteq \;\left\{\left|\;\frac{1}{n}\sum_{u=1}^{n}\sum_{i,j,k,l}\Delta_{i,j,k,l;u}\;1\left(\left|\frac{1}{12}\;d_{i,j,k,l}^{S}\right|>M\right)\;\right|>\epsilon /4\right\}\\ \; & \subseteq \;\left\{\left[\;\left|\frac{1}{12}\;d_{i,j,k,l}^{S}\right|>M\right]\;\mathrm{for}\mathrm{some}\;\;1\leq i,j,k,l\leq n\right\}, \end{array}$$

which implies

(A12) $$\begin{array}{c} \begin{array}{cc} \; & P\left(\left|\;\frac{1}{n}\sum_{u=1}^{n}\sum_{i,j,k,l}\Delta_{i,j,k,l;u}\;1\left(\left|\frac{1}{12}\;d_{i,j,k,l}^{S}\right|>M\right)-\rho_{02}^{*}\;\right|>\epsilon /2\right)\\ \; & \leq \;P\left(\left|\;\frac{1}{n}\sum_{u=1}^{n}\sum_{i,j,k,l}\Delta_{i,j,k,l;u}\;1\left(\left|\frac{1}{12}\;d_{i,j,k,l}^{S}\right|>M\right)\;\right|>\epsilon /4\right)\\ \; & \leq \;n^{4}\;P\left(\left|\frac{1}{12}\;d_{i,j,k,l}^{S}\right|>M\right)\;. \end{array} \end{array}$$

This is because, if $\left|\frac{1}{12}\;d_{i,j,k,l}^{S}\right|\leq M$ for all 1≤i,j,k,l≤n, then

$$n^{-1}\sum_{u=1}^{n}\sum_{i,j,k,l}\Delta_{i,j,k,l;u}\;1\left(\left|\frac{1}{12}\;d_{i,j,k,l}^{S}\right|>M\right)=0.$$

Under Condition (A1), Lemma 2 in the supplementary materials of [35] proves that there exists s>0 for which $E\left[\exp\left(s\;|\;d_{1234}^{S}|\right)\right]$ is finite. Using Markov’s inequality, we have

(A13) $$\begin{array}{c} \begin{array}{cc} P\left(\left|\frac{1}{12}\;d_{i,j,k,l}^{S}\right|>M\right)\; & \leq \;P\left(\;\exp\left(s\;\left|\frac{1}{12}\;d_{i,j,k,l}^{S}\right|\right)>\;\exp\left(sM\right)\right)\\ \; & \leq \;\exp\left(-sM\right)\;E\;\left[\exp\left(s\;\left|\frac{1}{12}\;d_{i,j,k,l}^{S}\right|\right)\right]\\ \; & \leq \;C_{2}\;\exp\left(-sM\right)\;\leq \;C_{2}\;\exp\left(-s_{1}\;n^{\gamma }\right)\; \end{array} \end{array}$$

for some positive constants $C_{2}$ and $s_{1}$, where the last line uses the fact that $M=c\;n^{\gamma }$. Combining (A12) and (A13), we have

(A14) $$\begin{array}{c} \mathrm{II}\;\leq \;C_{2}\;n^{4}\;\exp\left(-s_{1}\;n^{\gamma }\right)\;. \end{array}$$

Finally, combining (A7), (A9) and (A14), we obtain

$$\begin{array}{c} P\left(|{\hat{\rho }}^{\;*}-\rho_{0}^{*}|>\epsilon /2\right)\;\leq \;2\;\exp\left(-C_{1}n^{1-2\gamma }\epsilon^{2}\right)\;+\;C_{2}\;\;n^{4}\;\exp\left(-s_{1}n^{\gamma }\right)\; \end{array}$$

for some positive constants $\gamma ,C_{1},C_{2}$ and $s_{1}$. This completes the proof of the theorem. □

**Proof** **of** **Theorem** **2.** The first inequality in Theorem 2 simply follows by observing the fact that, for any generic random sequence ${\left\{X_{n}\right\}}_{n=1}^{\infty }$ and any ϵ>0,

$$\begin{array}{c} P(|X_{n}\left|>\epsilon \right)\;\leq \;P\left(\underset{n}{\sup}\;\left|X_{n}\right|>\epsilon \right) \end{array}$$

for all n≥1, which, in turn, implies

$$\begin{array}{c} \underset{n}{\sup}\;P(|X_{n}\left|>\epsilon \right)\;\leq \;P\left(\underset{n}{\sup}\left|X_{n}\right|>\epsilon \right)\;. \end{array}$$

The second inequality follows from union bound and Theorem 1. □

**Proof** **of** **Theorem** **3.** Denote by $E_{ab|S}$ the event that “an error occurs while testing for $X_{a}⫫X_{b}\;|\;X_{S}$" for a,b∈V and $S\in J_{a,b}^{m_{p_{n}}}$. Then,

(A15) $$\begin{array}{c} P\left(\;\mathrm{an}\mathrm{error}\mathrm{occurs}\mathrm{in}\mathrm{the}\mathrm{nonPC}\mathrm{algorithm}\;\right)\;\leq \;P\left(\underset{\begin{array}{c} a,b\;\in \;V\\ S\in J_{a,b}^{m_{p_{n}}} \end{array}}{⋃}E_{ab|S}\right)\;≲\;p_{n}^{m_{p_{n}}+2}\;P\left(E_{ab|S}\right)\; \end{array}$$

which is essentially due to the union bound. Now, we can write $E_{ab|S}=E_{ab|S}^{\;I}\cup E_{ab|S}^{\;\mathrm{II}}$, where

$$\begin{array}{cc} \; & \left(\mathrm{Type}I\mathrm{error}\right)\;E_{ab|S}^{\;I}\;:\;\left|{\hat{\rho }}_{ab|S}^{*}\right|>\xi_{\alpha }\;\mathrm{when}\;\;\rho_{0\;;\;ab|S}^{*}=0\\ \mathrm{and}\; & \left(\mathrm{Type}\mathrm{II}\mathrm{error}\right)\;E_{ab|S}^{\;\mathrm{II}}\;:\;\left|{\hat{\rho }}_{ab|S}^{*}\right|\leq \xi_{\alpha }\;\mathrm{when}\;\;\rho_{0\;;\;ab|S}^{*}>0\;. \end{array}$$

Then, by using triangle inequality,

(A16) $$\begin{array}{c} \begin{array}{cc} P(E_{ab|S}^{\;I})\; & =\;P(|\;{\hat{\rho }}_{ab|S}^{*}\;\left|>\xi_{\alpha }\right)\;=\;P\left(|\;{\hat{\rho }}_{ab|S}^{*}-\rho_{0\;;\;ab|S}^{*}+\rho_{0\;;\;ab|S}^{*}\;|>\xi_{\alpha }\right)\\ \; & \leq \;P\left(|\;{\hat{\rho }}_{ab|S}^{*}-\rho_{0\;;\;ab|S}^{*}\;|>\xi_{\alpha }-C_{max}\right)\\ \; & ≲\;2\;\exp\left(-A\;n^{1-2\gamma }{\left(\xi_{\alpha }-C_{max}\right)}^{2}\right)\;+\;n^{4}\;\exp\left(-Bn^{\gamma }\right)\; \end{array} \end{array}$$

for positive constants A,B and γ∈(0,1/4), where the last inequality follows from Theorem 2. Similarly, using the definition of $C_{min}$ and the identity |a|−|b|≤|a−b| for a,b∈R, we have

(A17) $$\begin{array}{c} \begin{array}{cc} P\left(E_{ab|S}^{\;\mathrm{II}}\right)\; & =\;P(|\;{\hat{\rho }}_{ab|S}^{*}\;|\leq \xi_{\alpha })\;=\;P(-|\;{\hat{\rho }}_{ab|S}^{*}\;\left|\geq -\xi_{\alpha }\right)\\ \; & =\;P(|\rho_{0\;;\;ab|S}^{*}|-|\;{\hat{\rho }}_{ab|S}^{*}|\geq |\rho_{0\;;\;ab|S}^{*}|-\xi_{\alpha })\\ \; & \leq \;P(|\rho_{0\;;\;ab|S}^{*}-{\hat{\rho }}_{ab|S}^{*}|\geq C_{min}-\xi_{\alpha })\\ \; & ≲\;2\;\exp\left(-A\;n^{1-2\gamma }{\left(\xi_{\alpha }-C_{min}\right)}^{2}\right)\;+\;n^{4}\;\exp\left(-Bn^{\gamma }\right)\;. \end{array} \end{array}$$

Again, the last inequality follows from Theorem 2. Combining Equations (A15)–(A17), we have

$$\begin{array}{cc} \; & \;P\;(\;\mathrm{an}\mathrm{error}\mathrm{occurs}\mathrm{in}\mathrm{the}\mathrm{nonPC}\mathrm{algorithm}\;)\\ \; & =\;O(\;p_{n}^{m_{p_{n}}+2}[\;2\;\exp\left(-A\;n^{1-2\gamma }{\left(\xi_{\alpha }-C_{max}\right)}^{2}\right)\;+\;2\;\exp\left(-A\;n^{1-2\gamma }{\left(\xi_{\alpha }-C_{min}\right)}^{2}\right]\\ \; & \;\;+\;n^{4}\exp\left(-B\;n^{\gamma }\right)])\\ \; & =\;o(1)\; \end{array}$$

where the last step follows from the fact that γ∈(0,1/4) and Assumption (A5). This implies that, as n→∞,

$$\begin{array}{cc} P\left({\hat{G}}_{\mathrm{skel},n}=G_{\mathrm{skel},n}\right)\; & =\;1\;-\;P\;(\;\mathrm{an}\mathrm{error}\mathrm{occurs}\mathrm{in}\mathrm{the}\mathrm{nonPC}\mathrm{algorithm}\;)\\ \; & \to \;1\;. \end{array}$$

□

**Proof** **of** **Theorem** **4.** The proof follows similar lines of the proof of Theorem 4.2 in [4], replacing Lemma 1.4 in their supplement by Theorem 2 in our paper. □
